# Synergistic anticancer effects of everolimus (RAD001) and Rhein on gastric cancer cells via phosphoinositide-3-kinase (PI3K)/protein kinase B (AKT)/mammalian target of rapamycin (mTOR) pathway

**DOI:** 10.1080/21655979.2021.2005988

**Published:** 2022-02-28

**Authors:** Feng Gao, Rui Li, Pei-Feng Wei, Li Ou, Min Li, Yang Bai, Wen-Jia Luo, Zheng Fan

**Affiliations:** a Teaching and Research Office of Chinese Pharmacy, The College of Pharmacy of Shaanxi University of Chinese Medicine, Xianyang, Shaanxi, China; b Department of Emergency, Affiliated Hospital of Shaanxi University of Chinese Medicine, Xianyang, Shaanxi, China; c The Office of Drug Clinical Trial Institution, The Second Affiliated Hospital of Shaanxi University of traditional Chinese Medicine, Xianyang, Shaanxi, China; d Department of Gastroenterology, Affiliated Hospital of Shaanxi University of Chinese Medicine, Xianyang712000, Shaanxi, China

**Keywords:** Gastric cancer, RAD001, Rhein, Pi3k/Akt/mTOR pathway

## Abstract

Everolimus (RAD001) is a mTOR inhibitor and is widely used for the treatment of gastric cancer (GC). Evidence suggests that Rhein has anticancer effect on GC. But the synergistic effect and mechanism of RAD001 and Rhein combination on GC is not clear. The current study aims to clarify the combination of RAD001 and Rhein in GC treatment. We found Rhein dose-dependently repressed MGC-803 cell viability (50% inhibition concentration (IC50) value = 94.26 μM). Rhein (80 μM) significantly suppressed GC cell proliferation and invasion. RAD001 dose-dependently repressed MGC-803 cells viability (IC50 value = 45.41 nM). The combination of Rhein and RAD001 repressed MGC-803 cells viability, invasion, and proliferation compared to the administration of Rhein or RAD001 alone. Protein levels of epithelial-mesenchymal transition (EMT)-related molecules E-cadherin, N-cadherin and Vimentin expressions were significantly affected by the combination of Rhein and RAD001. The combination of Rhein and RAD001 significantly facilitated cell apoptosis and up-regulated expressions of cell apoptosis and cycle-related protein p53, cyclin-dependent kinase 4 (CDK4) and cyclin D1 compared to the administration of Rhein or RAD001 alone. Moreover, the combination of Rhein and RAD001 repressed the expressions of phosphorylation-phosphoinositide-3-kinase (p-PI3K), p-protein kinase B (p-AKT) and p-mammalian target of rapamycin (p-mTOR). Finally, the combination of RAD001 and Rhein significantly decreased tumor weight and volume, suppressed the expressions of p-PI3K, p-Akt and p-mTOR, and repressed cell proliferation marker Ki-67 expression, which exerted synergistic cancer prevention in GC *in vivo*. Overall, the combination of Rhein and RAD001 exert synergistic cancer prevention in GC via PI3K/Akt/mTOR pathway.

## Introduction

Gastric cancer (GC) is a multifactorial-associated disease in the digestive system in the United States, which has an increasing number in new cases due to risk factors, including H. pylori infection, high salt intake, and smoking [1]. Most GC patients are diagnosed in advanced stage, which already show distant metastasis, such as that to the lung, bone and liver [2]. The progression of GC is complex which is related with the tumor cell proliferation, invasion, and migration [3]. Although some treatments have gained improvements in suppressing GC cell proliferation, invasion and migration [4,5], the five-year survival rate is not high [6]. Therefore, more effective treatments are needed for improving the survival rate of GC patients.

RAD001 (Everolimus), a mTOR inhibitor, is widely used for treating clear-cell renal cell carcinoma, breast cancer, and unresectable fibrolamellar carcinoma [7–9]. The PI3K/Akt/mTOR pathway plays an important role in modulating the biological functions of cancer cells, and PI3K/Akt/mTOR pathway activation is tightly related with GC progression [10,11]. Therefore, RAD001 is now commonly used to treat GC in clinic [12,13]. However, the dysregulation PI3K/Akt/mTOR pathway is related with chemoresistance resistance in patients with GC, and RAD001 resistance in GC will not effectively increase overall survival of GC patients [14,15].

Rhein is an anthraquinone component extracted from many medicinal herbs in traditional Chinese medicine, including polygonum multiflorum and rhubarb (Rheum rhabarbarum), which can exert anticancer functions via suppressing cancer cells proliferation and migration [16,17]. Recently, the combination of RAD001 and other drugs, such as capecitabine, mitogen-activated protein kinase kinase (MEK) inhibitor, and platinum, can increase overall survival of GC patients [18,19]. However, studies focused on the combination of RAD001 and Rhein in GC treatment are still lacked.

The current study assumes that RAD001 and Rhein combination has better inhibition effects on the activity of GC cells. We aim to investigate synergistic cancer prevention of RAD001 and Rhein in GC cell proliferation, growth and invasion, which may supply a rationale for RAD001 and Rhein combination in GC treatment.

## Materials and methods

### Cell culture and treatment

Human GC cells (MGC-803) were kept in our laboratory and were maintained in RPMI 1640 Medium (Gibco, CA, USA) supplemented with 10% fetal bovine serum (FBS; Gibco) at 37°C in 5% CO_2_ incubator [20].

Rhein was purchased from Sigma-Aldrich (MO, USA) with a purity ≥ 95%, and RAD001 (Everolimus) was purchased from Sigma-Aldrich. The drugs (Rhein and RAD001) were dissolved in saline.

### Measurement of IC50 values by MTT assay

MGC-803 cells were cultured in 96-well plates at a density of 2 × 10^3^ cells/well for 12 h. After the treatment of different concentrations of Rhein (0, 10, 20, 40, 80, 160 μM) and RAD001 (0, 2.5, 5, 10, 20, 40, 80, 160 nM) for 48 h, 10 μL/well MTT solution (Beyotime Biotechnology, Nantong, China) was added and cultured for 4 h. Formazan solution (100 μL/well) was added to MGC-803 cells and cultured for 4 h at 37°C. The absorbance was measured by a microplate reader (BioTek, Vermont, USA) at 570 nm. The IC50 values of RAD001 or Rhein against MGC-803 cells were calculated [21].

### Transwell assay

Transwell chamber (8 μm pore size, 24–well; Corning, NY, USA) was used to determine the invasion of MGC-803 cells [22]. After the treatment of Rhein and RAD001 for 48 h, MGC-803 cells (5 × 10^4^) were seeded in serum-free RPMI 1640 medium (Gibco) in the upper chamber pre-coated with matrigel (Corning). The lower chamber was added with RPMI 1640 medium supplemented with 10% FBS (Gibco). After cultured for 24 h, invaded MGC-803 cells in the lower chamber were collected and stained with 5% crystal violet solution. Images were obtained by a microscope (Nikon, Japan). Three images of ten random fields of each chamber were obtained, and number of invasive cells was counted.

### EdU staining

MGC-803 cell proliferation at different groups was determined using EdU staining [23]. MGC-803 cells at a density of 5 × 10^3^ cells/well were seeded in 96-well plates and incubated with 10 μM EdU (RiboBio, Guangzhou, China) for 120 min. MGC-803 cells were fixed with 4% paraformaldehyde for 15 min at 25°C. After stained with DAPI, images were acquired using a fluorescence microscope (Olympus, Japan).

### Flow cytometry

MGC-803 cells at different groups were collected in 1× Annexin V-FITC binding buffer (195 μL; Beyotime Biotechnology) to a density of 1 × 10^6^ cells/mL [24]. Then, Annexin V-FITC reagent (10 μL) was added to each tube that contained 1 × 10^6^ cells. Propidium iodide (PI; 10 μL) was added to MGC-803 cells. Cell apoptosis was distinguished by a flow cytometry (BD Biosciences, NJ, USA).

### TUNEL assay

MGC-803 cell apoptosis was detected by TUNEL assay kit (Beyotime Biotechnology). MGC-803 cells (2 × 10^6^ cells/mL) was collected and washed with 4% paraformaldehyde for 30 min at 25°C. Later, MGC-803 cells were added with 50 μL TUNEL solution at 37°C in the darkness for 60 min. MGC-803 cells can be analyzed by a fluorescence microscope.

### Western blotting

Protein was extracted from MGC-803 cells at different groups and tumor tissues using RIPA buffer containing PMSF. Proteins (50 µg) were resolved on 10% SDS-PAGE and electrotransferred on polyvinylidene fluoride membranes (Invitrogen, CA, USA). The primary antibodies used here were anti-PI3K, anti-p-PI3K, anti-AKT, anti-p-AKT, anti-mTOR, anti-p-mTOR, anti-E-cadherin, anti-N-cadherin, anti-Vimentin, anti-p53, anti-CDK4, anti-CyclinD1 or anti-beta-actin from Cell Signaling Technology or Abcam. Then, the membranes were incubated with secondary antibody and target proteins were analyzed by ImageLab software (Bio-Rad, CA, USA) [25].

### KEGG pathway enrichment analysis

The TCMIO database (http://tcmio.xielab.net/) is a commonly used database for providing information for Traditional Chinese Medicine [26]. We can generate herb-ingredient-target networks online, and KEGG pathway enrichment analysis for target polygonum multiflorum is available. *P* < 0.05 indicated statistically significant.

### Xenograft model

Female CB-17 SCID mice (specific-pathogen-free, five-week-old) were purchased from Beijing Charles River Co. and maintained in sterile condition. MGC-803 cells (1 × 10^7^) were inoculated subcutaneously into each CB-17 SCID mouse. Mice were divided into control, RAD001, Rhein, RAD001+ Rhein groups, with six mice in each group. Mice received Rhein (60 mg/kg) or/and RAD001 (5 mg/kg) by oral administration. Tumor volumes were measured every five days as follows: volume = length×width^2^/2. All mice were sacrificed 28 days later, and tumor xenografts were excised and weighed. All studies were supported by Animal Care and Research Committee of The college of Pharmacy of Shaanxi University of Chinese Medicine.

### Immunohistochemical (IHC) staining

Tumor tissues were fixed and paraffin embedded, and 4 μm-thick sections were collected from paraffin specimens [27]. The sections were heated in citrate buffer and immunostained with anti-Ki-67. Ki-67 expression was evaluated under a microscope (Olympus, Japan).

### Statistical analysis

Data were expressed as mean ± SD. Differences between groups were performed by Student’s t test. Differences among multiple groups were performed by One-way analysis of variance (ANOVA) for multiple comparisons followed by Bonferroni post hoc test. Synergistic and comparative RAD001/Rhein analyses on GC cell experiment were statistically calculated with R value. P value <0.05 was identified as significant.

## Results

We aim to investigate synergistic cancer prevention and the underlying mechanism of RAD001 and Rhein in MGC-803 cells proliferation and invasion *in vitro* and *in vivo*. We calculated IC50 value for Rhein and RAD001 dose response, and finish-dose selection for Rhein and RAD001. We found Rhein and RAD001 combination repressed MGC-803 cells proliferation and invasion *in vitro* through PI3K/Akt/mTOR pathway and verified our hypothesis *in vivo*.

### Rhein suppressed the proliferation and invasion of MGC-803 cells

**Figure 1.**
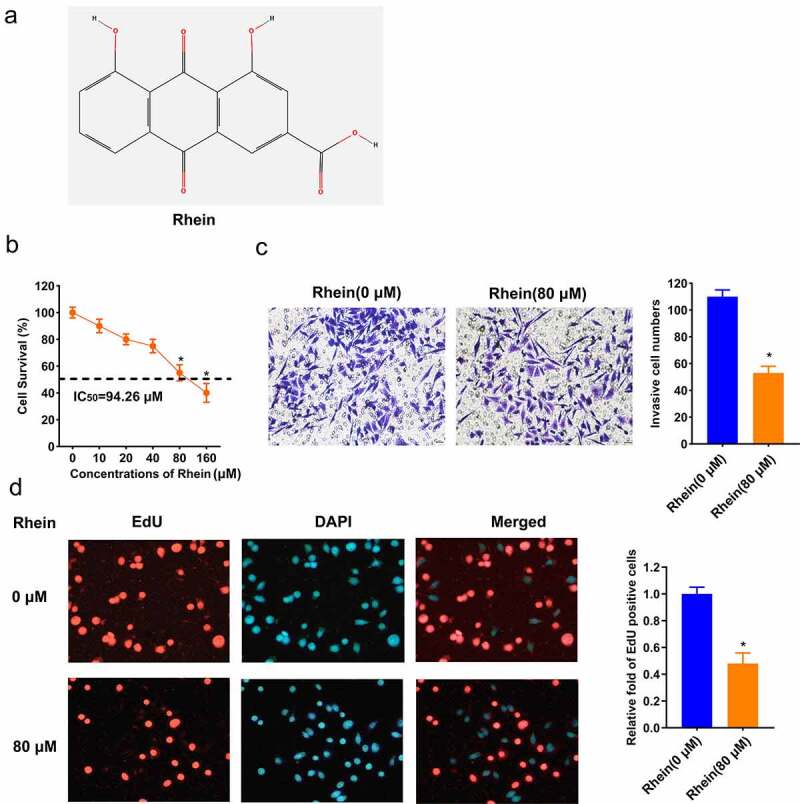
Rhein suppressed MGC-803 cell proliferation and invasion. A. structure of Rhein. B. MGC-803 cells were treated with 0, 10, 20, 40, 60, 80, 160 μM Rhein. after 48 hours, IC50 value for Rhein dose response was calculated by MTT assay. C. MGC-803 cells were treated with 0, 80 μM Rhein, MGC-803 cell invasion was detected using transwell assay. D. EdU-staining was applied to detect MGC-803 cell proliferation. EdU-positive cells of MGC-803 cells after 0, 80 μM Rhein treatment for 48 h (×100). EdU was in red. DAPI was in blue. *p < 0.05.

The structure of Rhein is shown in Figure 1(a). A dose-dependent inhibition of MGC-803 cell survival was observed by different concentrations of Rhein treatment, and IC50 value for Rhein was 94.26 μM (Figure 1(b)). So, 80 μM of Rhein without causing significant toxicity was chosen for further experiments. We observed that MGC-803 cell survival was significantly repressed at a concentration of 80 μM Rhein (Figure 1(b)). Rhein (80 μM) significantly repressed the number of invasive MGC-803 cells compared to Rhein (0 μM) group (Figure 1(c)). EdU assay showed that EdU-positive cells was significantly inhibited in Rhein (80 μM) group compared to Rhein (0 μM) group (Figure 1(d)). So, the effective response of Rhein (80 μM) can reduce the activity of MGC-803 cells.

### Synergistic inhibition impacts of Rhein and RAD001 on MGC-803 cell proliferation and invasion

**Figure 2.**
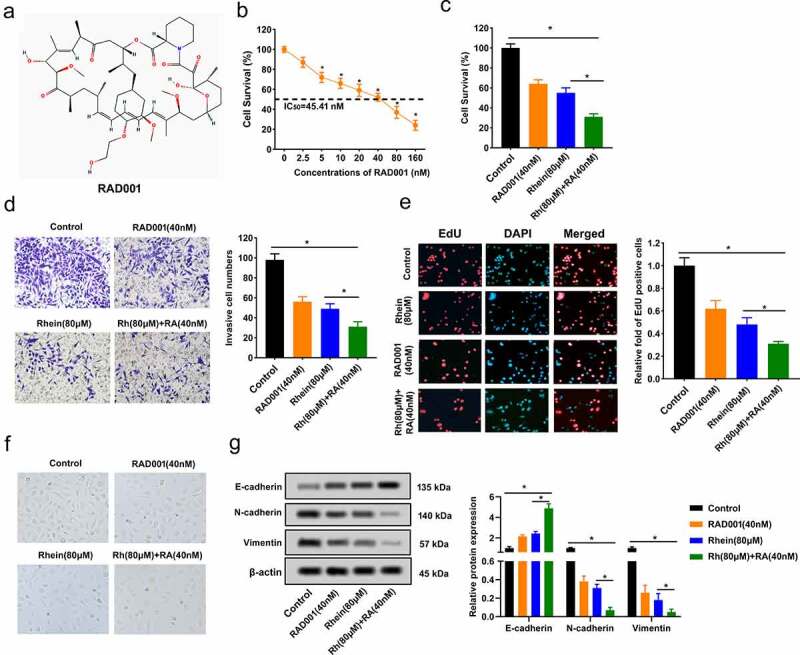
Synergistic inhibition impacts of Rhein and RAD001 on MGC-803 cell proliferation and invasion. A. structure of RAD001. B. MGC-803 cells were treated with 0, 2.5, 5, 10, 20, 40, 80, 160 nM RAD001. after 48 hours, IC50 value for RAD001 dose response was calculated using MTT. C. Rhein (80 μM) and RAD001 (40 nM) were used for MGC-803 cells, then cell viability was measured using MTT. D. Rhein (80 μM) and RAD001 (40 nM) were used for MGC-803 cells, then MGC-803 cell invasion was detected using transwell. E. EdU-staining was applied to detect MGC-803 cell proliferation. EdU-positive cells of MGC-803 cells after 80 μM Rhein or 40 nM RAD001 treatment for 48 h (×100). F. Light microscope images of MGC-803 cells were observed. G. protein levels of EMT-related molecules E-cadherin, N-cadherin, vimentin were measured using Western blotting. *p < 0.05.

RAD001 structure is shown in Figure 2(a). It showed a dose-dependent inhibition of cell survival in MGC-803 cells, and IC50 value for RAD001 was 45.41 nM (Figure 2(b)). So, a concentration of 40 nM without causing significant toxicity was chosen to treat MGC-803 cells for further experiments. We further observed the effect of Rhein (80 μM) and RAD001 (40 nM) combination on MGC-803 cell survival. MGC-803 cell survival was significantly inhibited by treatment of Rhein and RAD001 (Figure 2(c)), MGC-803 invasive cells were significantly repressed in Rhein and RAD001 combination group compared to the other three groups (Figure 2(d)), and EdU assay showed EdU-positive cells was significantly decreased in Rhein and RAD001 combination group compared to the other three groups (Figure 2(e)). As shown in Figure 2(f), light microscope images of MGC-803 cells in control, the combination of Rhein and RAD001 group, and the administration of Rhein or RAD001 alone groups were observed. Moreover, protein levels of EMT-related molecule E-cadherin was significantly up-regulated in Rhein (80 μM) and RAD001 (40 nM) group compared to the other three groups, whereas N-cadherin and Vimentin expressions were significantly down-regulated in Rhein (80 μM) and RAD001 (40 nM) group compared to the other three groups (Figure 2(g)).

### Synergistic promotion impacts of Rhein and RAD001 on MGC-803 cell apoptosis

**Figure 3.**
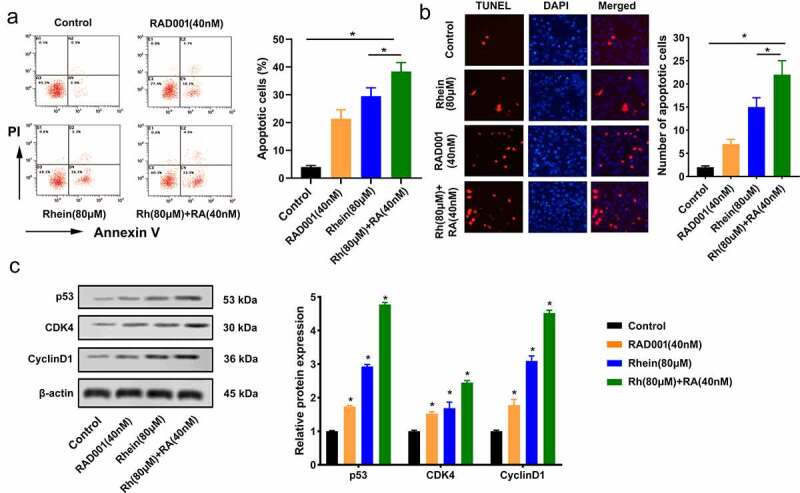
Synergistic promotion impacts of Rhein and RAD001 on MGC-803 cell apoptosis. Rhein (80 μM) and RAD001 (40 nM) were used to treat MGC-803 cells. A. Rhein and RAD001 combination in MGC-803 cell apoptosis was detected using flow cytometery. B. Rhein and RAD001 combination in MGC-803 cell apoptosis was detected by TUNEL assay. C. The effect of Rhein and RAD001 combination on p53, CDK4 and Cyclin D1expressions was measured using Western blotting. *p < 0.05.

Rhein and RAD001 together significantly promoted MGC-803 cell apoptosis compared to the other three groups (Figure 3(a)). TUNEL assay further showed that the treatment of Rhein and RAD001 together significantly promoted MGC-803 cell apoptosis compared to the other three groups (Figure 3(b)). Moreover, compared to the other three groups, the treatment of Rhein and RAD001 together significantly up-regulated the protein expressions of p53, CDK4 and Cyclin D1 (Figure 3(c)).

### Synergistic inhibition effects of Rhein and RAD001 on PI3K/AKT/mTOR pathway

**Figure 4.**
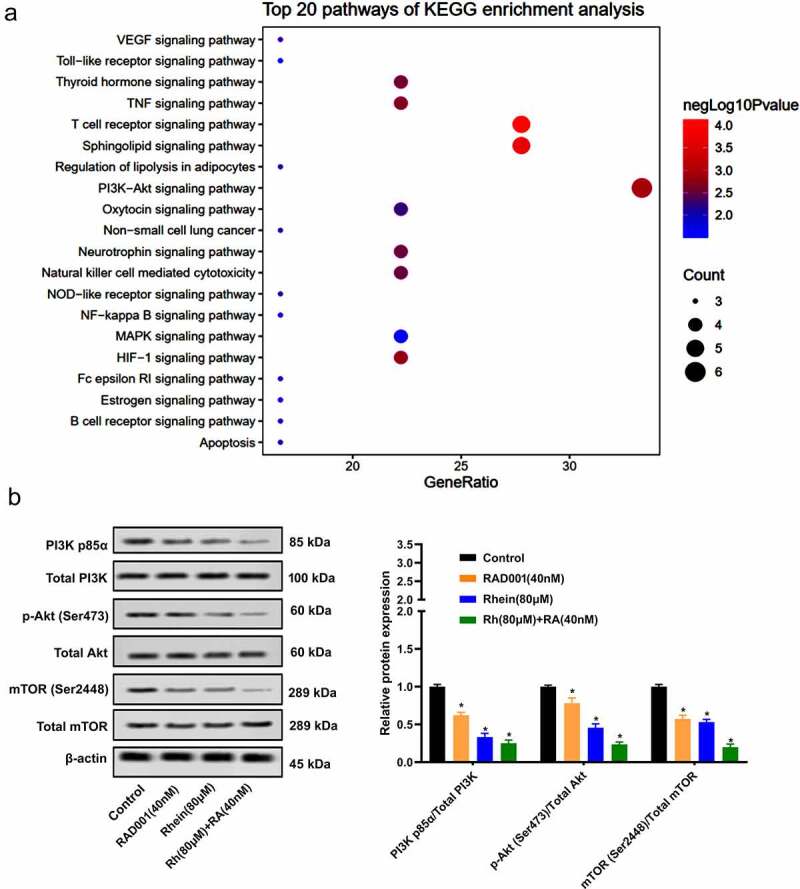
Synergistic inhibition impacts of Rhein and RAD001 on PI3K/AKT/mTOR pathway. A. KEGG pathway enrichment analysis associated with the target polygonum multiflorum. B. The effect of Rhein and RAD001 combination on PI3K/AKT/mTOR pathway proteins expressions was measured using Western blotting. *p < 0.05.

A KEGG pathway enrichment analysis associated with target Polygonum multiflorum was performed using TCMIO database, and the results are shown in Figure 4(a). According to the KEGG pathways in Figure 4(a), Polygonum-multiflorum-targeted pathways are enriched in the following, e.g., PI3K-Akt signaling pathway, T cell receptor signaling pathway, sphingolipid signaling pathway, TNF signaling pathway, MAPK signaling pathway. The target polygonum multiflorum was mainly enriched in PI3K-Akt signaling pathway, which gained the highest score (Figure 4(a)). The scores of other signaling pathways were much lower than the score of PI3K-Akt signaling pathway, so we chose PI3K-Akt signaling pathway for this study. In addition, compared to the other three groups, the treatment of Rhein and RAD001 together significantly down-regulated protein levels of phosphorylation of PI3K (p-PI3K), p-AKT, and p-mTOR (Figure 4(b)). These results indicated that the synergistic inhibition effects of Rhein and RAD001 on the activation of PI3K/AKT/mTOR pathway.

### *Synergistic inhibition impacts of Rhein and RAD001 on MGC-803 cell proliferation* in vivo

**Figure 5.**
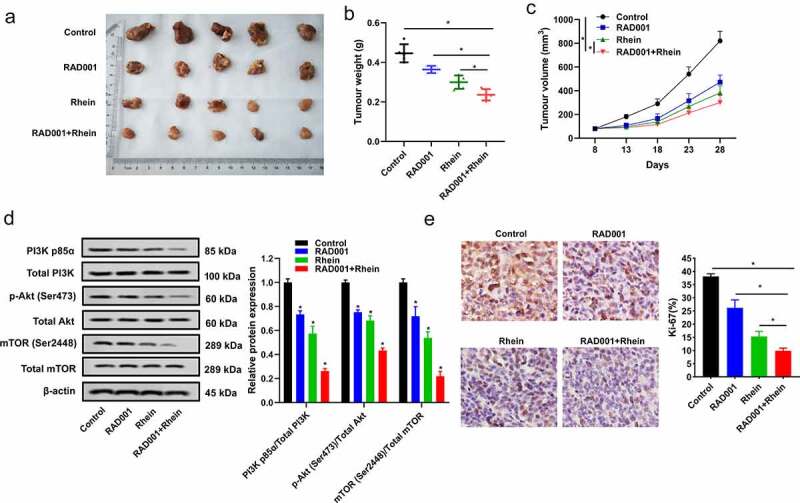
Synergistic inhibition effects of Rhein and RAD001 on MGC-803 cell proliferation *in vivo*. A-C. Mice received Rhein (60 mg/kg) and RAD001 (5 mg/kg) by oral administration. Tumor weight and tumor volume were detected at each group. D. The expressions of PI3K/AKT/mTOR pathway proteins were detected using Western blotting. E. Immunohistochemical (IHC) staining of tumor cell proliferation marker Ki-67 expression. *p < 0.05.

To verify whether Rhein and RAD001 could synergistically repress MGC-803 cell proliferation *in vivo*, Rhein (60 mg/kg) and RAD001 (5 mg/kg) were used for the observation of the anticancer effects on MGC-803 cell xenograft mouse model. The combination of Rhein and RAD001 significantly repressed tumor weight, and decreased tumor volume at day 18 to day 28 than control group, indicating they delayed xenograft growth (Figure 5(a-c)). In addition, compared with the other three groups, the combination of Rhein and RAD001 significantly down-regulated p-PI3K, p-AKT and p-mTOR protein expressions (Figure 5(d)), indicating Rhein and RAD001 combination inhibited PI3K/AKT/mTOR pathway activation *in vivo*. Furthermore, combination of Rhein and RAD001 significantly decreased tumor cell proliferation marker Ki-67 expression by IHC staining (Figure 5(e)).

## Discussion

Rhein, an anthraquinone component extracted from herbs in traditional Chinese medicine such as polygonum multiflorum and rhubarb, has been used for over 1000 years in China for alleviating inflammation due to its strong curative effects and few side effects [28]. Recent studies have implicated Rhein exerts anticancer effects on a variety of cancers through suppressing cancer cell growth and invasion, and promoting cancer cell apoptosis [17,29,30]. Li et al. have found that Rhein induces human GC cell SGC-7901 apoptosis in a dose-dependent manner [31]. However, the effects of Rhein on GC cell growth, proliferation, and invasion are not clarified. We found that Rhein repressed GC cell viability in a dose-dependent manner, and inhibited GC cell invasion. With about 47% cell viability reduction, a significant anticancer impact was attained when the concentration of Rhein at 80 μM.

Combination treatment is considered as a promising therapeutic method in GC [32]. Several studies have provided evidences that the combination of RAD001 and other drugs (Asp-Glu-Ala-Asp box helicase 5, celecoxib, cyclophosphamide) can improve GC cell sensitivity to RAD001, which exert better anticancer effects on GC [12,14,33]. In previous researches, Rhein can sensitize human pancreatic cancer cells and human colorectal cancer cells to erlotinib [34,35]. The combination of Rhein and doxorubicin can promote the treatment impact of doxorubicin in ovarian cancer cells and reduce drug resistance of ovarian cancer cells [36]. Rhein and atezolizumab combination has stronger therapeutic effects on mouse breast cancer via repressing the growth and promoting the apoptosis than atezolizumab therapy group [37]. These researches provide evidences that Rhein can sensitize a variety of cancer cells to anticancer drugs. However, whether Rhein can enhance anticancer effects of RAD001 on GC is still not clear. We assumed that Rhein might enhance the antitumor effect of RAD001 on GC through suppressing GC cell proliferation and invasion, and induce apoptosis, which had better inhibitory effects on GC than RAD001 alone. Finally, we found that Rhein and RAD001 combination has stronger anticancer effects on GC cells through inhibiting the cancer cell invasion and proliferation than the administration of Rhein or RAD001 alone, which will enrich the literature.

According to previous reports, RAD001 can induce cell cycle arrest at G0/G1 phase and promote GC cell apoptosis [12,38]. In addition, Rhein has antiapoptotic effects on GC cells [31], can induce cell cycle arrest at the G0/G1 phase, S phase or G2 phase in human colorectal cancer cells and liver cancer cells, as well as reduce cell cycle-related protein levels [35,39,40]. However, the effect of Rhein and RAD001 combination on GC cell apoptosis is not clear. We showed that Rhein and RAD001 combination remarkably facilitated GC cell apoptosis than the administration of Rhein or RAD001 alone, and upregulated the expressions of cell apoptosis-related protein p53. These findings first confirmed the effect of Rhein and RAD001 combination on GC apoptosis.

PI3K/Akt/mTOR pathway activation promotes the invasion and migration of GC cells [41,42]. p-PI3K could activate Akt to form p-Akt, and PI3K inhibitor LY294002 could repress the expressions of p-PI3K, p-Akt, and p-mTOR in GC cells, as well as induce the apoptosis and suppress the proliferation of GC cells [43]. mTOR pathway is modulated by PI3K/Akt pathway, and suppressing PI3K/Akt pathway can repress the expression of p-PI3K and p-Akt, thereby inhibiting p-mTOR expression in GC [44]. Importantly, p-mTOR can lead to worse prognosis and shorter overall survival rate in GC [45]. Researchers also have shown evidences that PI3K/Akt/mTOR pathway is a key pathway regulated by Rhein to exert its anticancer effect on GC [46,47]. Moreover, PI3K/Akt/mTOR pathway modulates RAD001 in epithelial mesenchymal transition phenotype, apoptosis, and cell cycle arrest of GC cells [48]. We further confirmed that PI3K/Akt/mTOR pathway activation can be inhibited by Rhein and RAD001 combination than the administration of Rhein or RAD001 alone. These results indicated that PI3K/AKT/mTOR pathway involves in RAD001 and Rhein exerted synergistic anticancer effects on GC cells.

RAD001 can inhibit tumor growth of GC cell xenografts [12]. However, whether RAD001 and Rhein combination can exert synergistic anticancer effects on GC *in vivo* is still not known. In this *in vivo* experiment, we found that the combination of Rhein and RAD001 significantly reduced tumor weight and volume, repressed PI3K/AKT/mTOR pathway activation, and decreased tumor cell proliferation marker Ki-67 expression than the administration of Rhein or RAD001 alone, which indicated RAD001 and Rhein combination exerted synergistic cancer prevention on GC *in vivo* xenograft model.

Limitations: Whether GC cell autophagy participates in the process of synergistic cancer prevention of RAD001 and Rhein combination is not investigated. In addition, the effect of Rhein on RAD001-resistant GC cells is not explored. We will focus on the effect of RAD001 and Rhein combination on GC cell autophagy, and clarify if there are microRNAs or other signaling pathways involved in the regulation of RAD001 and Rhein combination in GC.

## Conclusion

Our data calculate IC50 value for Rhein and RAD001 dose response, and finish-dose selection for Rhein and RAD001. In addition, our data suggest RAD001 and Rhein combination exerts their synergistic cancer prevention through suppressing GC cell proliferation and invasion, and promoting GC cell apoptosis *in vitro*, and in vivo experiments verified Rhein and RAD001 combination repressed tumor growth and PI3K/AKT/mTOR pathway activation.
